# SIRT1-dependent restoration of NAD+ homeostasis after increased extracellular NAD+ exposure

**DOI:** 10.1016/j.jbc.2021.100855

**Published:** 2021-06-11

**Authors:** Daniela Buonvicino, Giuseppe Ranieri, Maria Pittelli, Andrea Lapucci, Stefania Bragliola, Alberto Chiarugi

**Affiliations:** 1 Department of Health Sciences, Section of Clinical Pharmacology and Oncology, University of Florence, Florence, Italy; 2 Corporate Pre-Clinical R&D, Chiesi Farmaceutici S.p.A., Parma, Italy

**Keywords:** NAD+, homeostasis, bioenergetics, sirtuin 1 (SIRT1), transport, ADO, adenosine, AMPCP, adenosine 5′-(α,β-methylene)diphosphate, CD38, ADP-ribosyl hydrolase/cyclase, CD73, ecto-5′-nucleotidase, eNAD+, extracellular NAD+, ENT, equilibrative nucleoside transporter, iNAD+, intracellular NAD+, NAM, nicotinamide, NAMPT, nicotinamide phosphoribosyl transferase, NMN, nicotinamide adenine mononucleotide, NMNAT, nicotinamide mononucleotide adenylyl transferases, NR, nicotinamide riboside, NRK1, nicotinamide riboside kinase, PARPs, polyADP-ribose polymerases, SIRTs, sirtuins, Slc12a8, solute carrier family 12 member 8, TFs, transcription factors

## Abstract

In the last several years, NAD+ supplementation has emerged as an innovative and safe therapeutic strategy for a wide spectrum of disorders, including diabetes and neuropathy. However, critical questions remain as to how NAD+ and its precursors are taken up by cells, as well as the effects of long-lasting intracellular NAD+ (iNAD+) increases. Here, we investigated the kinetics of iNAD+ levels in different cell types challenged with prolonged exposure to extracellular NAD+ (eNAD+). Surprisingly, we found that after the initial increase, iNAD+ contents decreased back to control levels (iNAD+ resetting). Focusing our attention on HeLa cells, we found that oxygen and ATP consumption occurred with similar temporal kinetics after eNAD+ exposure. Using [^3^H]NAD+ and [^14^C]NAD+, we determined that NAD+ resetting was not due to increased dinucleotide extrusion but rather due to reduced uptake of cleaved NAD+ products. Indeed, eNAD+ exposure reduced the expression of the ecto-5′-nucleotidase CD73, the nicotinamide adenine mononucleotide transporter solute carrier family 12 member 8, and the nicotinamide riboside kinase. Interestingly, silencing the NAD+-sensor enzyme sirtuin 1 prevented eNAD+-dependent transcriptional repression of ecto-5′-nucleotidase, solute carrier family 12 member 8, and nicotinamide riboside kinase, as well as iNAD+ resetting. Our findings provide the first evidence for a sirtuin 1–mediated homeostatic response aimed at maintaining physiological iNAD+ levels in conditions of excess eNAD+ availability. These data may be of relevance for therapies designed to support the NAD+ metabolome *via* extracellular supplementation of the dinucleotide or its precursors.

In the last several years, our understanding of the NAD+ metabolome extended from that of a mere electron-carrying cofactor for oxidation–reduction reactions to an essential enzymatic substrate involved in key signaling pathways (1) such as those brought about by polyADP-ribose polymerases (PARPs), monoADP-ribosyltransferases, sirtuins (SIRTs), and the ADP-ribosyl hydrolase/cyclase (CD38). These enzymes play pivotal roles in DNA repair, gene expression, cell cycle regulation, and calcium signaling (2). In keeping with the important roles of these functions in disease pathogenesis, a large body of evidence shows that impairment of NAD+ availability, due to either reduced biosynthesis or increased consumption, promotes aging (3, 4), diabetes (5), obesity (6), and neurodegeneration (7). On this basis, maintenance of intracellular NAD+ (iNAD+) homeostasis may have pleiotropic therapeutic effects. This is well in keeping with experimental evidence that boosting iNAD+ contents with its metabolic precursors such as nicotinamide mononucleotide (NMN) and nicotinamide riboside (NR) is of therapeutic relevance in a wide number of disparate disease models, such as diabetes and neuropathy (5, 8, 9, 10, 11, 12, 13, 14). The therapeutic potential of NAD+ and its precursors has been also investigated in several clinical trials (15, 16, 17, 18, 19, 20). More recently, small-molecule activators of nicotinamide phosphoribosyl transferase (NAMPT), the rate-limiting enzyme of the NAD+ rescue pathway, have been proposed as an additional NAD+ boosting strategy for cytoprotection (21, 22). On the contrary, pharmacological restriction of iNAD+ availability with specific and potent NAMPT inhibitors may represent an innovative and promising anticancer strategy by impairing the dinucleotide's support to energy metabolism and proliferation (23, 24, 25, 26, 27, 28).

Despite the impressive progress made in the field of NAD+ signaling and its involvement in disease pathogenesis, whether/how NAD+ or its precursors are taken up by the different cell types and to what extent this contributes to NAD+ homeostasis is still a matter of debate. Indeed, whereas bacteria (29), yeast (30), and mitochondria (31) are endowed with specific membrane NAD+ carriers, the plasma membrane NAD+ transporter has yet to be identified. This is in spite of several studies providing evidence that NAD+ crosses the plasma membrane of mammalian cells uncleaved (32, 33, 34, 35, 36, 37). Additional contributions, however, show that extracellular NAD+ (eNAD+) can be degraded by the ecto-5′-nucleotidase (CD73) into NMN and NR (38, 39), with the former being hydrolyzed by CD38 into nicotinamide (NAM) and ribose 5-phosphate (40) or transported intracellularly by the solute carrier family 12 member 8 (Slc12a8) carrier (41), and the latter possibly entering the cell *via* nucleoside transporters (42, 43, 44).

Regardless of the mechanisms underpinning NAD+ uptake, various studies report that the increased availability of eNAD+, both *in vitro* and *in vivo*, prompts metabolic and signaling responses that, as a whole, improve bioenergetics and resistance to stress (32, 34, 35, 36, 45, 46, 47, 48, 49, 50). In this regard, it is worth noting that the temporal kinetics of these perturbations are currently unknown. Indeed, whether cells can prompt compensatory responses aimed at resetting iNAD+ homeostasis once the latter has been altered by increased extracellular availability of the dinucleotide waits to be investigated. This is an essential point in light of the therapeutic potential ascribed to various NAD+ boosting strategies in disorders typically characterized by a chronic pattern of progression that, by definition, would necessitate continuous exposure to NAD+ or its metabolic precursors.

On this basis, in the present study, we attempted to understand whether and how iNAD+ homeostasis changes over time in cells challenged with a prolonged increase of eNAD+ availability.

## Results

### Effects of prolonged eNAD+ exposure on cellular metabolism

**Figure 1:**
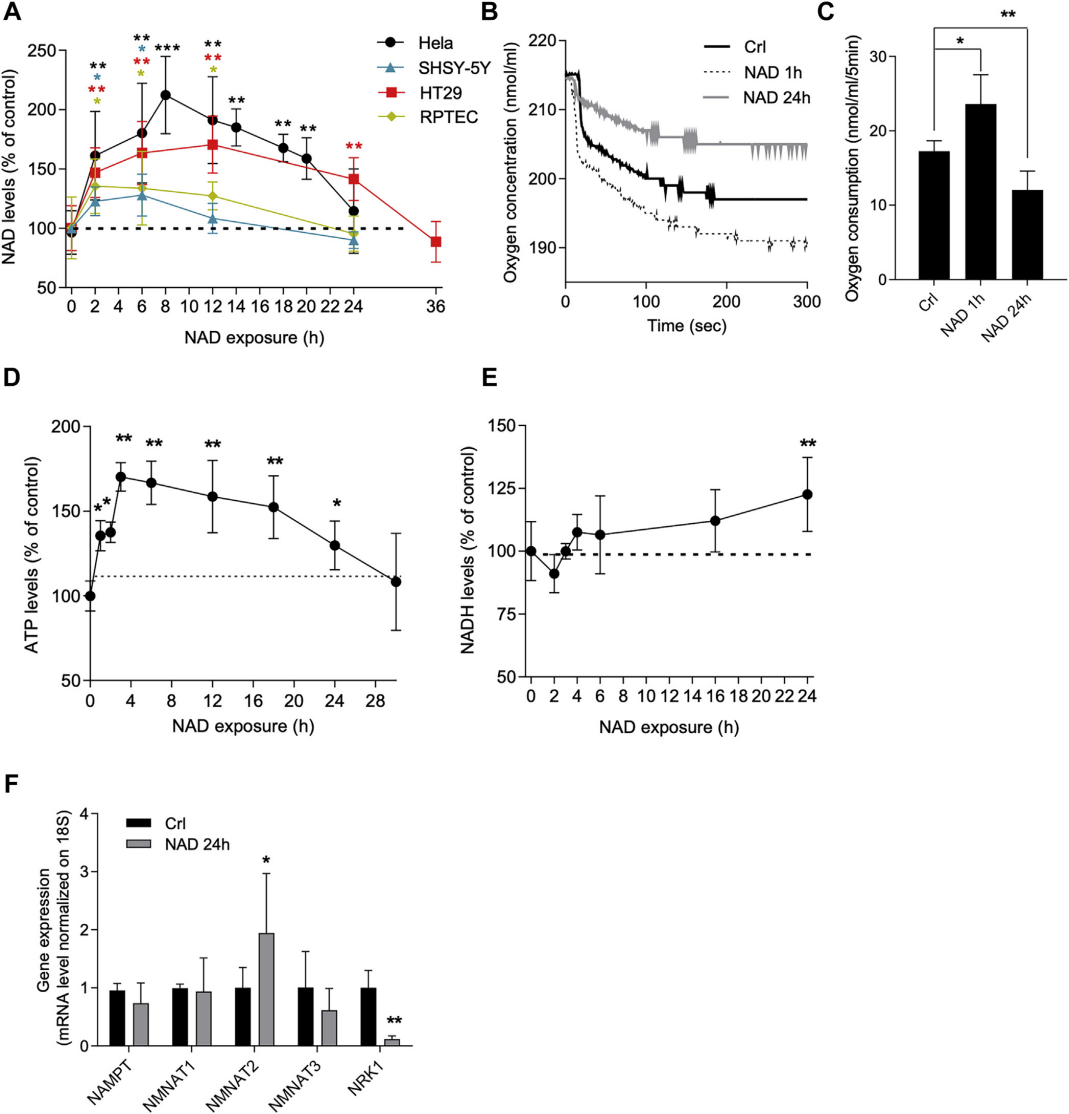
**Effects of prolonged eNAD+ exposure on cellular metabolism in HeLa cells.** Effects of 1 mM eNAD+ on iNAD+ contents over time in different cell types (*A*). Representative (*B*) and quantitative (*C*) analysis of oxygen consumption rate by HeLa cells exposed for 1 h or 24 h to eNAD+ (1 mM). Effects of 1 mM eNAD+ on ATP contents over time in HeLa cells (100% equal 72 ± 2.3 nmol/mg protein) (*D*). Effects of 1 mM extracellular NAD+ on intracellular NADH contents over time in HeLa cells (*E*). Effects of 1 mM NAD+ on *NAMPT*, *NMNAT1*-*3*, and *NRK1* transcripts in HeLa cells after 24-h incubation (*F*). Each column/point represents the mean ± SD of four experiments in triplicate. ∗*p* < 0.05, ∗∗*p* < 0.01, ∗∗∗*p* < 0.001 *versus* Crl. ANOVA and Tukey's post hoc test were used. eNAD+, extracellular NAD+; iNAD+, intracellular NAD+; NAMPT, nicotinamide phosphoribosyl transferase; NMNATs, nicotinamide mononucleotide adenylyl transferases; NRK1, nicotinamide riboside kinase.

We previously reported that iNAD+ contents increase upon a brief exposure of HeLa cells to eNAD+ and that this increase confers significant cytoprotection from apoptosis triggered by staurosporine, C2 ceramide, or N-methyl-N′-nitro-N-nitrosoguanidine (32). These findings are in line with numerous reports also showing cytoprotective properties of eNAD+ (32, 34, 35, 36, 45, 46, 47, 48, 49, 50). To our knowledge, however, the effects of prolonged exposure of resting cells to eNAD+ have never been investigated. To address this issue, we evaluated the effects of 1 mM eNAD+ on iNAD+ contents in HeLa cells during a 24-h incubation. We found that iNAD+ contents increased from 9.2 ± 1.3 to 19.5 ± 2.4 nmol/mg protein at 8 h and then, surprisingly, linearly decreased over time to return to basal levels after 24 h (Fig. 1*A*). Notably, the same results were obtained when cells were exposed to lower eNAD+ concentrations (Fig. S1*A*). Furthermore, to rule out that this finding was specific for HeLa cells, we also evaluated the effects of eNAD+ over time in SHSY-5Y (neuroblastoma), HT29 (colorectal adenocarcinoma), and renal proximal tubule epithelial cells and found similar kinetics of iNAD+ contents upon NAD+ exposure (Fig. 1*A*). Given that eNAD+ exposure increases both cellular oxygen consumption and ATP content (32), we next attempted to understand whether these two bioenergetic parameters also showed a biphasic response upon a prolonged challenge to eNAD+. Indeed, the augmented oxygen consumption present in cells exposed to 1 mM NAD+ for 1 h vanished at 24 h, reaching values lower than those of resting cells (Fig. 1, *B* and *C*). In keeping with this, we found that ATP contents promptly increased in cells exposed to eNAD+, reaching a maximum at 3 h (from 72 ± 2.3 to 122.4 ± 8.4 nmol/mg protein) and then showed a slow decrease over time reaching the basal level after 30-h incubation (Fig. 1*D*). Notably, we found that cells exposed to lower eNAD+ concentration presented a lower increase of ATP contents (Fig. S1*B*) and oxygen consumption (Fig. S1*C*), which underlies that the changes of these metabolic parameters are linked to changes in NAD contents. We also found a small increase in the ADP pool probably because of a contribution of eNAD+ to the adenylate pool (Fig. S1*D*). The unexpected return of iNAD+ contents to control levels during eNAD+ exposure hereinafter referred to as “iNAD+ resetting,” prompted us to first hypothesize that it was merely due to its reduction to NADH. We, therefore, measured the cellular contents of NADH over time during eNAD+ exposure and found that they increased by 22.59 ± 3% after 24 h (Fig. 1*E*). Given that the extent of this increase was not consistent with that of NAD+ resetting (equaling a reduction of 112.28 ± 31% of peak values) (Fig. 1*A*), we reasoned that mechanisms in addition to NADH formation should be involved. Given that NAMPT and nicotinamide mononucleotide adenylyl transferases (NMNATs) play a key role in the maintenance of iNAD+, we hypothesized that changes in their expression levels could contribute to NAD+ resetting. We found, however, that transcript levels for NAMPT, NMNAT1, and NMNAT3 did not change and those for NMNAT2 almost doubled, whereas those of nicotinamide riboside kinase (NRK1) decreased by 9-fold in cells exposed to eNAD+ for 24 h (Fig. 1*F*).

### Prolonged eNAD+ exposure reduces eNAD+ uptake

**Figure 2:**
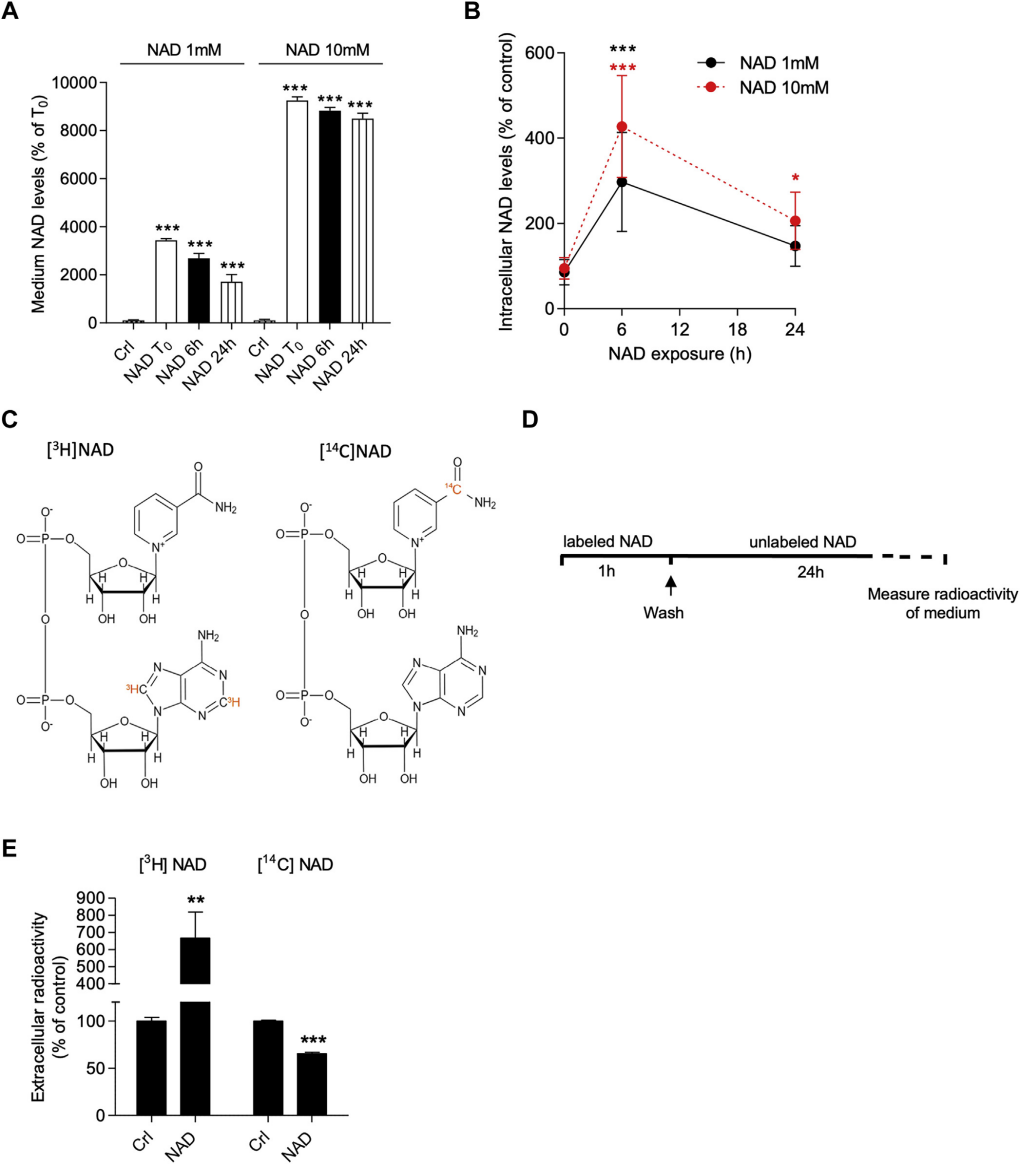
**Prolonged eNAD+ exposure does not induce NAD extrusion.** Effects of 1 or 10 mM eNAD on NAD contents in the medium (*A*) and the cells (*B*) after 0- (T_0_), 6-, and 24-h eNAD exposure. Chemical drawing of labeled [^3^H]NAD and [^14^C]NAD (*C*). Experimental scheme to evaluate NAD+ extrusion (*D*). Effects of unlabeled NAD+ (1 mM, 24 h) on release of radioactivity in the medium by cells preincubated for 1 h with [^3^H]NAD+ (500 pM) or [^14^C]NAD+ (500 pM) (*E*). Each column/point represents the mean ± SD of three experiments in triplicate. ∗∗*p* < 0.01, and ∗∗∗ *p* < 0.001 *versus* Crl. ANOVA and Tukey's post hoc test were used. eNAD+, extracellular NAD+.

In an attempt to identify additional mechanisms underlying iNAD+ resetting, we hypothesized involvement of the NAD+-consuming enzymes PARPs or/and mono (ADP-ribose) polymerases. We found, however, that a high concentration of two different PARP inhibitors PJ34 (10 μM) (51) and 6(5H)-phenanthridinone (30 μM) (52), as well as the inhibitor of mono (ADP-ribose) polymerases novobiocin (100 μM) (53), did not affect the kinetic of NAD+ resetting (data not shown). Next, to rule out the possibility that iNAD resetting might be due to eNAD depletion over time, we analyzed the NAD contents after 24 h in the medium. We found a progressive NAD depletion over time in the medium; however, eNAD contents did not reach control levels after 24-h incubation (Fig. 2*A*). Moreover, we performed a new experiment adopting 10 instead of 1 mM eNAD+ and found that a similar iNAD resetting occurred despite a lack of eNAD depletion over time, ruling out that iNAD+ resetting is related to eNAD depletion (Fig. 2, *A* and *B*). As an alternative hypothesis, we investigated whether NAD+ resetting might be due to extracellular extrusion of the dinucleotide. To this end, we measured the extent of radioactivity extrusion by cells preloaded for 1 h with adenine-labeled [^3^H]NAD+ (Fig. 2*C*) and then exposed or not to unlabeled eNAD+ for 24 h (Fig. 2*D*). Interestingly, we found increased radioactivity in the medium of eNAD+-exposed cells (Fig. 2*E*), thereby suggesting that extrusion indeed contributes to NAD+ resetting. Still, to make sure that the dinucleotide is extruded intact, we repeated the experiment with NAM-labeled [^14^C]NAD+ (Fig. 2*C*). Surprisingly, we found exactly the opposite result, that is, a decreased amount of radioactivity extruded in the medium of eNAD+-exposed cells (Fig. 2*E*). These findings on the one hand indicated that NAD+ resetting is not due to a mere extrusion of NAD+ and on the other hand that radioactivity observed in the medium is related to NAD+-cleaved products.

**Figure 3:**
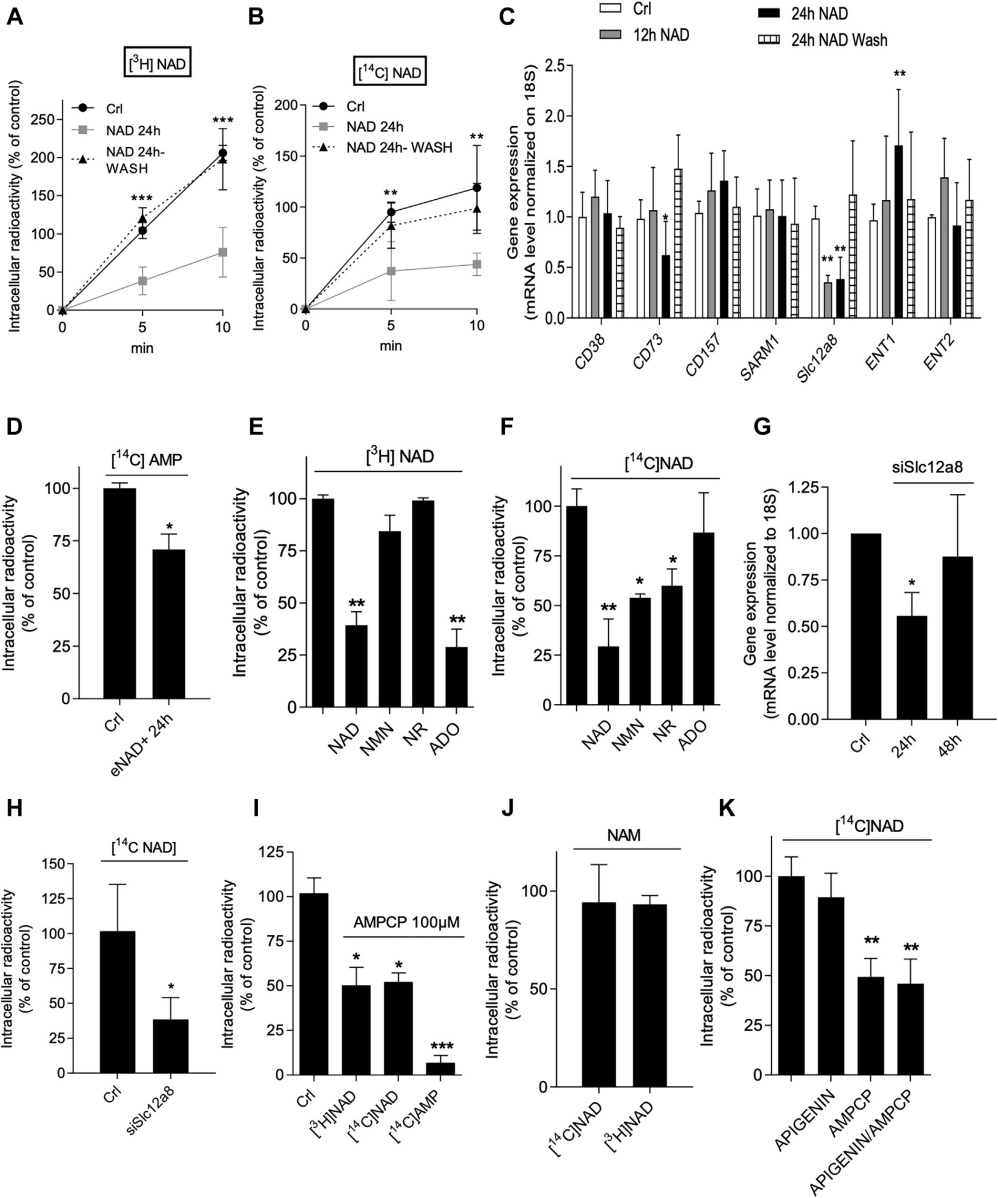
**Prolonged eNAD+ exposure reduces NAD+ uptake by HeLa cells.** Effects of unlabeled NAD+ (1 mM, 24 h) and its subsequential withdrawal (24 h) on time-dependent uptake of 500 pM [^3^H]NAD+ (*A*) or 500 pM [^14^C]NAD+ (*B*). Effects of 1 mM extracellular NAD+ on NAD+-degrading enzymes and transporters transcript levels in cells after 12- and 24-h NAD+ incubation and its subsequential withdrawal (24 h) (*C*). Effects of unlabeled eNAD+ (1 mM, 24 h) on radioactivity uptake of cells exposed to [^14^C]AMP (500 pM) (*D*). Effects of NAD+ and NAD+ derivatives (each one 1 mM) on uptake of 500 pM [^3^H]NAD+ (*E*) or [^14^C]NAD+ (*F*). Effects of Slc12a8 silencing on its transcript levels after 24 h and 48 h (*G*). Effects of 48-h Slc12a8 silencing on uptake of 500 pM [^14^C]NAD+ (*H*). Effects of AMPCP (100 μM/30 min) on 500 pM [^3^H]NAD+, [^14^C]NAD+, or [^14^C]AMP (*I*). Effects of NAM (1 mM) on 500 pM [^3^H]NAD+ or [^14^C]NAD+ (*J*). Effects of apigenin (100 μM), AMPCP (100 μM), or both on uptake of 500 pM [^14^C]NAD+ (*K*). Each column/point represents the mean ± SD of four experiments in triplicate. ∗*p* < 0.05, ∗∗*p* < 0.01, and ∗∗∗ *p* < 0.001 *versus* Crl. ANOVA and Tukey's post hoc test were used. eNAD+, extracellular NAD+; Slc12a8, solute carrier family 12 member 8; AMPCP, adenosine 5′-(α,β-methylene)diphosphate.

We next moved on to check whether reduced eNAD+ uptake contributes to NAD+ resetting. We, therefore, evaluated whether the uptake of radioactivity originating from [^3^H]NAD+ or [^14^C]NAD+ differed in cells exposed or not to eNAD+. Interestingly, we found that the uptake of the radioactivity of both labeled NAD+ moieties was lower in cells exposed to eNAD+ for 24 h and returned to control levels upon 24 h eNAD+ washing (Fig. 3, *A* and *B*). The fact that radioactivity was measured in cells that already reset NAD+ content rules out that the reduced uptake might be due to high intracellular dinucleotide levels. These findings, therefore, suggested that mechanisms responsible for NAD+ uptake are altered upon prolonged eNAD+ exposure. As mentioned above, experimental evidence indicates that NAD+ is taken up by the plasma membrane both intact (32, 33, 35, 36) and upon hydrolysis (42, 43, 44). In this regard, although a putative plasma membrane NAD+ transporter is yet to be identified, several membrane carriers and enzymes have been involved in the uptake of NAD+-cleaved products (42, 43, 44). Specifically, eNAD+ is cleaved into NMN and AMP by CD73 (38, 39) and by CD203a (also known as PC-1) (54), or converted into ADP ribose and NAM by CD38 and its paralog CD157 (40, 55, 56). We, therefore, analyzed the transcript levels of both ectoenzymes in cells challenged with a 12- or 24-h exposure to eNAD+ and found a significant reduction of mRNAs of CD73 after 24 h and no effect on those of CD38 and CD157, whereas CD203a transcript levels were not detectable (Fig. 3*C*). To confirm that reduction of CD73 transcripts was functionally relevant, we measured intracellular adenosine (ADO) contents upon extracellular [^14^C]AMP exposure as a prototypical indirect index of CD73 activity (57). In keeping with the PCR data, we found that the increase of intracellular radioactivity was lower in eNAD+-exposed cells than the controls (Fig. 3*D*). Notably, a complete restoration of CD73 transcript levels occurred upon eNAD+ washing (Fig. 3*C*). We also evaluated the effect of eNAD+ on transcript levels of SARM1, an important NADase that could play a role in NAD degradation (58). We found that eNAD+ exposure did not alter mRNA levels of SARM1 over time (Fig. 3*C*). Conversely, we found that transcript levels of the NMN transporter Slc12a8 were already reduced in cells after 12-h eNAD+ exposure and similar to those of CD73 recovered upon washing (Fig. 3*C*). In light of the putative role of plasma membrane equilibrative nucleoside transporters (ENTs) in eNAD+ uptake (34), we also evaluated whether eNAD+ exposure changed mRNA levels of ENT1 and ENT2. We found that eNAD+ prompted substantial increases of ENT1 mRNAs, having no effects on those of ENT2 (Fig. 3*C*). We also investigated whether gene expression changes observed in eNAD+-treated cells also occurred in cells exposed to NAD-cleaved products. Interestingly, we found that among NRK1, CD73, ENT1, and Slc12a8, only the expression of the latter is reduced by 24-h NMN or ADO exposure, whereas NR had no effects on analyzed genes (Fig. S1*E*).

In light of the role of CD73 and Slc12a8 in the uptake of NAD+-cleaved products, our findings suggested that the reduced expression of both proteins could contribute to iNAD+ resetting. To corroborate this hypothesis, we again took advantage of [^3^H]-adenine–labeled and [^14^C]-NAM–labeled NAD+ and checked whether radioactivity uptake changed in the presence of unlabeled eNAD+ or its presumed cleaved products. We found that NAD+ and ADO reduced the uptake of radioactivity originating from [^3^H]NAD+ (Fig. 3*E*), whereas NAD+, NMN, and NR reduced the uptake of radioactivity originating from [^14^C]NAD+ (Fig. 3*F*). Notably, silencing of NMN transporter Slc12a8 reduced the uptake of radioactivity when NAM-labeled [^14^C]NAD+ was added extracellularly (Fig. 3, *G* and *H*), indicating a contribution of this transporter on uptake of NAD+. To evaluate the contribution of serum enzymes involved in eNAD+ hydrolysis (44), we performed experiments studying the uptake of radioactivity from labeled NAD+ moieties without serum. As shown in Figure S2, *A* and *B*, we found that radioactivity uptake is not affected by the absence of serum.

These findings suggest that, at least in part, eNAD+ is cleaved extracellularly and the degradation products are taken up by the plasma membrane through different routes in keeping with prior work (38, 39). To further corroborate these results, we evaluated the effects of the CD73 inhibitor adenosine 5′-(α,β-methylene)diphosphate (AMPCP) (59) on eNAD+ uptake. We found that AMPCP was able to reduce the uptake of radioactivity when both [^3^H]NAD+ and [^14^C]NAD+ were added extracellularly (Fig. 3*I*), thereby confirming the key role of eNAD+ hydrolysis by the ectoenzyme for subsequent uptake of degradation products. It is worth noting that we used AMPCP at a concentration capable of reducing the uptake of radioactivity originating from [^14^C]AMP by 94 ± 3% (Fig. 3*I*). Given that this radioactivity is related to the uptake of [^14^C]adenosine formed extracellularly by CD73 (57), the latter finding suggested that under our experimental setting AMPCP prompted an almost complete inhibition of the ectoenzyme. On this basis, we reasoned that the cellular radioactivity accumulating in cells exposed to labeled NAD+ in the presence of AMPCP (Fig. 3*I*) is CD73 independent. Accordingly, we found that prolonged exposure to AMPCP affected the increase of iNAD+ in the early time points when cells were exposed to unlabeled eNAD+ (Fig. S2*C*). We then evaluated the CD73-independent metabolism of eNAD+ *via* the NAM-forming CD38. We found that the addition of a molar excess of extracellular NAM, used as a product inhibitor of CD38, did not alter radioactivity uptake when cells were exposed either to [^3^H]NAD+ or to [^14^C]NAD+ (Fig. 3*J*). In keeping with this, the CD38 inhibitor apigenin (60) did not reduce the uptake of radioactivity originating from [^14^C]NAD+ (Fig. 3*K*). Hence, the CD73-independent uptake of radioactivity in cells exposed to labeled NAD+ might be ascribed to intact NAD+ transportation. Accordingly, we found that NAMPT or NRK1 silencing did not prevent iNAD+ increase after eNAD+ exposure (Fig. S2, *D* and *E*), supporting the hypothesis that, at least in part, NAD+ was transported intact.

Collectively, these findings indicate that increased iNAD+ content due to prolonged eNAD+ exposure triggers a complex cascade of transcriptional events involving plasma membrane eNAD+-metabolizing enzymes and transporters aimed at reestablishing NAD+ homeostasis.

### SIRT1 activity prompts iNAD+ resetting

**Figure 4:**
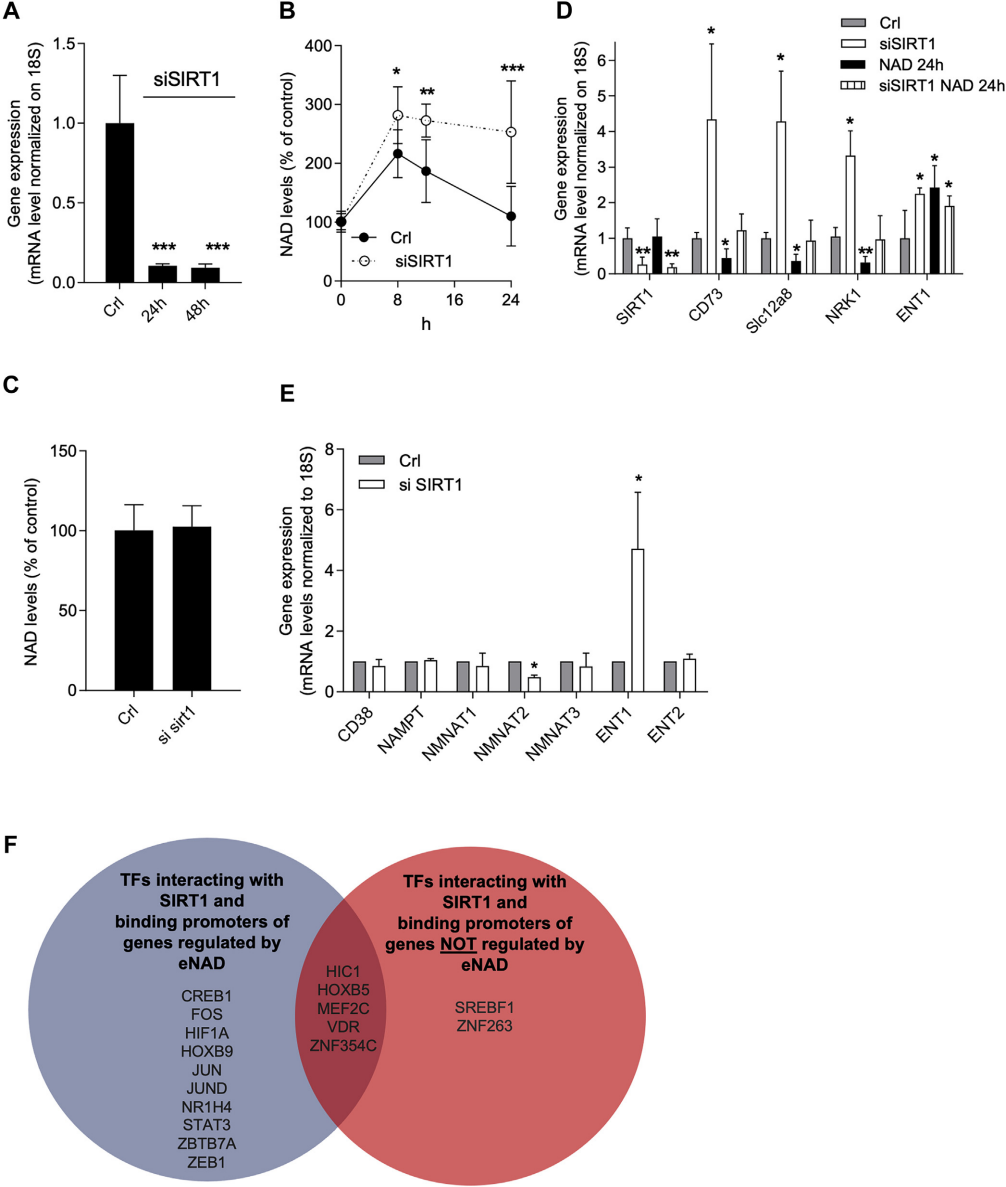
**SIRT1 silencing prevents iNAD+ resetting under prolonged eNAD+ exposure in HeLa cells.** Effects of SIRT1 silencing on its transcript levels after 24 h and 48 h (*A*). Effects of SIRT1 silencing on NAD+ content of cells exposed to extracellular NAD+ (1 mM) (*B*). Effects of 48-h SIRT1 silencing on intracellular NAD contents in the resting condition (*C*). Effects of SIRT1 silencing in presence or not of NAD+ (1 mM, 24 h) on SIRT1, CD73, Slc12a8, NRK1, and ENT1 transcript levels (*D*). Effects of SIRT1 silencing on CD38, NAMPT, NMNAT1-3, and ENT 1/2 transcript levels (*E*). In silico analysis of the transcription factors binding the promoters of different genes evaluated in cells exposed to eNAD+. Venn diagram of SIRT1-interacting transcription factors binding promoters of genes whose expression is altered (*blue*) and unaltered (*red*) during NAD+ resetting (*F*). Each column/point represents the mean ± SD of four experiments in triplicate. ∗*p* < 0.05, ∗∗*p* < 0.01, and ∗∗∗ *p* < 0.001 *versus* Crl. ANOVA and Tukey's post hoc test were used. CD38, ADP-ribosyl hydrolase/cyclase; CD73, ecto-5′-nucleotidase; eNAD+, extracellular NAD+; ENT, equilibrative nucleoside transporter; iNAD+, intracellular NAD+; NAMPT, nicotinamide phosphoribosyl transferase; NMNAT, nicotinamide mononucleotide adenylyl transferase; NRK1, nicotinamide riboside kinase; SIRT1, sirtuin 1; Slc12a8, solute carrier family 12 member 8.

To further understand the molecular mechanisms underlying iNAD+ resetting, we focused our attention on SIRTs. The latter are NAD+-dependent deacetylases with a key transcription-regulating function (61). Among them, SIRT1 is the most extensively studied and is involved in the regulation of various metabolic processes in response to changes in iNAD+ availability (62). We hypothesized that changes in SIRT1 activity due to increased substrate availability concur to regulate the expression of enzymes/carriers involved in iNAD+ resetting. To verify this hypothesis, we suppressed SIRT1 expression approximately by 90% by means of siRNA (Fig. 4*A*). Next, we set up a silencing protocol in which cells were exposed or not to siRNA for 48 h and to eNAD+ for the last 24 h. Remarkably, as shown in Figure 4*B*, the increase in iNAD+ at 8 h was significantly higher in SIRT1-silenced cells than that occurring in cells proficient for SIRT1. More importantly, silencing of SIRT1 abrogated NAD+ resetting (Fig. 4*B*). To rule out that silencing SIRT1 could increase basal iNAD+ contents and the latter might be at least in part responsible for preventing iNAD+ resetting, we measured iNAD+ contents under basal conditions (*i.e.*, without eNAD+ exposure) in resting and SIRT1-silenced cells. As shown in Figure 4*C*, SIRT1 silencing did not affect iNAD+ content in basal condition. In keeping with a key role of CD73 and Slc12a8 transcriptional suppression in iNAD+ resetting, silencing of SIRT1 prevented downregulation of both transcripts in eNAD+-exposed cells (Fig. 4*D*). SIRT1 silencing also sufficed to prompt CD73 and Slc12a8 mRNA increases in control cells (Fig. 4*D*), consistent with SIRT1-dependent constitutive suppression of CD73 and Slc12a8 transcription. Similarly, also NRK1 transcriptional suppression induced by prolonged eNAD+ exposure (Fig. 1*F*) was reverted by SIRT1 silencing, and, again, its basal transcript levels increased in SIRT1-silenced cells (Fig. 4*D*). Notably, we found that genes that did not affect during eNAD+ exposure (NAMPT, NMNAT1, NMNAT3, ENT2, CD38) were not regulated by SIRT1 silencing, contrarily NMNAT2 and ENT1, whose levels were changed by NAD+ exposure, were affected by SIRT1 silencing (Fig. 4*E*). Collectively, these data on the one hand highlight the role of SIRT1 in regulating the expression of genes involved in eNAD+ metabolism and uptake and on the other hand to its transcriptional regulating ability as a central mechanism of NAD+ resetting. To corroborate this assumption, we conducted an in silico analysis of the transcription factors (TFs) known to bind the promoters of the various genes we evaluated in cells exposed to eNAD+. Specifically, we divided the genes into two groups, that is, those whose expression is affected during NAD+ resetting (CD73, Slc12a8, ENT1, NRK1, NMNAT2) and those that do not undergo transcriptional changes (NAMPT, NMNAT1, NMNAT3, ENT2, CD38). Using the Ciiider software (63) (developed to identify TF DNA-binding elements) and the database Biological General Repository for Interaction Datasets (www.thebiogrid.org, a repository for protein–protein interaction), for each gene, we then evaluated the number of TFs binding to the corresponding promoter and known to interact with SIRT1. Interestingly, as shown in Figure 4*F*, the number of SIRT1-regulated TFs was 15 for genes whose expression changed during NAD+ resetting and seven for those showing no transcriptional changes. The higher number of SIRT1-regulated TFs binding to promoters of genes altered during eNAD+ exposure supports the hypothesis that SIRT1 contributes to the transcriptional changes during iNAD+ resetting.

## Discussion

In light of the consolidated role of iNAD+ availability in counteracting pathogenesis of numerous disorders, extracellular supply of the dinucleotide and its precursors received great attention as a safe and innovative therapeutic strategy. In this scenario, the present study demonstrates that cells bring about a SIRT1-dependent homeostatic response aimed at restoring physiological iNAD+ contents. SIRT1 being a key sensor of iNAD+ content and transcriptional regulator makes sense that the enzyme is centrally poised to regulate the NAD+ resetting response.

A key finding of our study is that upon eNAD+ exposure, cells undergo transient iNAD+ increase followed by an exact resetting to control levels. Of note, this occurs despite the continuous presence of high extracellular dinucleotide concentrations. Intrigued by the underlying molecular mechanism, we set up experiments to understand whether resetting was due to NAD+ consumption and/or extrusion. As far as consumption is concerned, according to the literature (64), PARP-1 is the sole enzyme that can prompt an extent of NAD+ consumption consistent with the quantitative aspect of NAD+ resetting (*i.e.*, an amount of NAD+ corresponding to almost 100% of its basal content, see Fig. 1*A*). However, two potent PARP inhibitors proved unable to affect NAD+ resetting, in keeping with the apparent lack of triggers prompting PARP-1 activation in cells with increased iNAD+ contents. Our findings also rule out the possibility that NAD+ resetting is due to dinucleotide extrusion. Indeed, although cells loaded with [^3^H]NAD+ increase extrusion of radioactivity upon eNAD+ exposure, the opposite occurs in those loaded with [^14^C]NAD+. This is not consistent with the extrusion hypothesis as the mechanisms are responsible for NAD+ resetting. Rather, these findings, together with evidence of induction of the ENT1 in eNAD+-exposed cells (Fig. 3*C*), indicate that a reduction in intracellular ADO compromises the adenylate pool for the resynthesis of NAD+. Although this mechanism may well underlie iNAD+ resetting, the additional finding that ATP contents are increased rather than reduced during iNAD+ resetting rules out this hypothesis. Also, the increased availability of ATP in eNAD+-exposed cells suggests that iNAD+ resetting cannot be ascribed to a reduced dinucleotide resynthesis through the ATP-dependent NAMPT and NMNATs.

The reduction in uptake of radioactivity originating both from [^3^H]NAD+ and [^14^C]NAD+ when cells are pre-exposed to eNAD+ suggests that reduced eNAD+ uptake contributes to iNAD+ resetting. Specifically, data indicate that, at least in part, resetting is due to diminished eNAD+ hydrolysis and ensuing uptake of related products. In this regard, prior works report the hydrolysis of eNAD+ by CD73 (38, 39). Our data are in keeping with this claim by showing that while extracellular ADO affects the uptake of the radioactivity of [^3^H]-adenine-labeled NAD+, NR and NMN reduce that of [^14^C]-NAM–labeled NAD+. Taken together, these findings suggest that eNAD+ is cleaved extracellularly into adenine- and NAM-containing moieties that are taken up by at least two different routes, such as ENTs and Slc12a8, respectively. Of note, the inability of NR to reduce the uptake of radioactivity originating from [^3^H]NAD+ (that is [^3^H]adenosine) (Fig. 3*D*) also suggests that, at least under our experimental conditions, NR does not compete with ADO to cross the plasma membrane *via* ENTs. Conversely, the ability of the CD73 inhibitor AMPCP to reduce the uptake of radioactivity originating from both [^3^H]NAD+ and [^14^C]NAD+ indicates the key functional role of CD73 in eNAD+ hydrolysis and subsequent increase of iNAD+. In this regard, the reduced CD73 expression levels, as well as its activity in cells challenged with eNAD+, points to these transcriptional repressions as a central event in NAD+ resetting. Evidence that, akin to CD73, the expression of both Slc12a8 (a specific NMN transporter (41, 65)) and NRK1 (the enzyme feeding NR into the NAD+ resynthesis pathway (66)) diminishes upon eNAD+ exposure provides additional mechanisms underpinning iNAD+ resetting. Of note, we report that Slc12a8 silencing reduced [^14^C]-NAM–labeled NAD+ uptake, confirming the role of this transporter in NAD+ uptake and its contribution to restoring NAD+ contents to control levels. Admittedly, additional molecular mechanisms might concur to reestablishing iNAD+ content upon prolonged eNAD+ exposure. We provide here, however, the first evidence that, among these mechanisms, SIRT1 is a key player of the iNAD+ resetting response. In particular, our data suggest that the increased availability of iNAD+ in eNAD+-exposed cells promotes SIRT1 activity that, in turn, prompts CD73, Slc12a8, and NRK1 transcriptional repression. This compromises the efficiency of the molecular machinery responsible for eNAD+-induced iNAD+ increase, thereby contributing to iNAD+ resetting. The key role of SIRT1 in iNAD+ resetting is well in keeping with the ability of the enzyme to adjust its activity according to the intracellular availability of NAD+, as well as with its epigenetic functions (62). This interpretation is also in line with our in silico analysis showing that those genes whose expression changes during iNAD+ resetting have some SIRT1-interacting TFs double that of genes not affected by eNAD+ exposure. It is also worth noting that silencing of SIRT1 suffices to prompt a 4-fold increase of CD73 and Slc12a8 transcripts in resting cells, thereby indicating a constitutive role of the enzyme in repressing transcription of these genes. Of note, we found that SIRT1 silencing prompts transcriptional changes only toward genes that are affected by eNAD+ exposure, thereby underlying the correlation between NAD+ contents and transcriptional regulation by SIRT1.

Regardless of the role of CD73 and Slc12a8 in eNAD+ uptake, our data also suggest that eNAD+ can cross the plasma membrane uncleaved. Indeed, in the presence of an extracellular concentration of AMPCP leading to an almost complete CD73 inhibition (see Fig. 2*H*), eNAD+ can still prompt significant iNAD+ increases. Furthermore, silencing of NAMPT or NRK1 did not reduce the iNAD+ increase under eNAD exposure, suggesting that part of NAD+ was not cleaved. This is in keeping with prior findings on the ability of eNAD+ to trigger iNAD+ increases without evidence for extracellular hydrolysis (32, 33, 35, 36). Admittedly, even if our assumption is supported by prior and present experimental evidence, it is not consistent with the ability of SIRT1 silencing to prevent iNAD+ resetting by reducing CD73 and Slc12a8 NMN transporter. This apparent inconsistency might be reconciled if even the expression of the yet to be identified plasma membrane NAD+ transporter was negatively regulated by SIRT1. However, only NAD+ transporter identification will confirm this hypothesis.

Our study presents some limitations. First, we report total iNAD+ concentration, without specifying in which compartment NAD+ increase occurred; this limit may be the subject of future studies. Second, because under physiological conditions, in mammalian serum, NAD+ circulates in the low micromolar range (0.1–0.5 μM), NAD+ concentrations used in our study are doubtless high. However, our findings lay the basis for a finely tuned signaling pathway able to sense derangements of NAD+ homeostasis and prompt resetting of physiological conditions. Third, we emphasize that even if our findings provide evidence for unprecedented molecular machinery responsible for resetting cellular NAD+ content upon an initial increase, they do not clarify how iNAD+ decreases during NAD+ resetting and reaches exactly the original control values. Indeed, the SIRT1-activated feedback mechanism impinging on CD73, Slc12a8, and NRK1 expression, in principle, should only counteract further iNAD+ increases, somehow freezing the cell in a cytoplasmic milieu containing an increased NAD+ content. In other words, mechanisms responsible for reducing iNAD+ to the initial basal levels, once the entrance apparatus has been impaired, are still unknown.

From a theoretical point of view, in light of the pleiotropic and critical functions of NAD+ signaling (DNA repair, transcription, Ca^2+^ homeostasis, energetic metabolism, mitochondrial dynamics, etc.), it makes sense that evolution provided eukaryotic cells with feedback molecular machinery aimed at counteracting derangements of NAD+ availability that might severely compromise cellular homeostasis. Given the great interest in the NAD+ boosting approach, evaluating this regulatory mechanism could prevent saturation phenomena *in vivo* and help identify the therapeutic dosing regimen.

In conclusion, the present study furthers our understanding of the complex network of events that regulate iNAD+ content. Finally, it suggests that the therapeutic potential of the different NAD+-boosting strategies (67) might be significantly reduced in those tissues in which the NAD+-resetting response is functionally relevant. Of course, NAD+-supplementation strategies may still be of therapeutic relevance in conditions of iNAD+ deficiency.

## Experimental procedures

### Cell culture

HeLa, primary renal proximal tubule epithelial, SH-SY5Y, and HT29 human cells were obtained from American Type Culture Collection. Cells were grown in Dulbecco's modified Eagle's medium (DMEM) containing 25 mM glucose and supplemented with 2 mM glutamine, 1 mM pyruvate, 10% fetal bovine serum, and antibiotics. Cultures were brought to 50 to 70% confluence and exposed to different compounds. NAM dinucleotide (NAD+), NMN, NAM, and ADO were purchased by Sigma-Aldrich. NR was synthesized as described by Yang *et al*. (68). NAD+, NMN, NR, NAM, and ADO were dissolved in culture media. Apigenin was dissolved in DMSO (Sigma-Aldrich). PJ34, 6(5H)-phenanthridinone, AMPCP, and novobiocin were dissolved in water (Sigma-Aldrich). All results are expressed as the percentage of the control (untreated cells); each sample was normalized by the protein content.

### NAD+, NADH, ADP, and ATP measurement

NAD+ and NADH contents were quantified through an enzymatic cycling procedure as reported (32). Briefly, to measure NAD+ contents, cells grown in a 48-well plate were killed with 1 M 50 μl of HClO_4_ and then neutralized with an equal volume of 1 N KOH. After the addition of 100 μl of bicine (100 mM), 100 μl of the cell extract was mixed with an equal volume of the bicine buffer containing ethanol, 3-(4,5-dimethylthiazol-2-yl)-2,5-diphenyltetrazolium, phenazine ethosulfate, and alcohol dehydrogenase. The mixture was kept at room temperature for 10 min, and then, absorbance at 550 nm was measured (VICTOR3, PerkinElmer). To measure intracellular NADH contents, cells grown in a 24-well plate were killed with 1 N 80 μl of KOH and then heated to 60 °C for 30 min. After the addiction of 500 μl of bicine (100 mM), 100 μl of the cell extract was mixed with an equal volume of the bicine buffer containing ethanol, 3-(4,5-dimethylthiazol-2-yl)-2,5-diphenyltetrazolium, phenazine ethosulfate, and alcohol dehydrogenase and measured as described above. ADP contents were quantified in 0.25 N HCl cell extracts (from 6-well plate) by HPLC using a Supelco 25-cm column (5 μm), mobile phase K_2_HPO_4_ 0.1 M, 1% acetonitrile, 10 mM tetrabutylammonium bromide, pH 6.9, and UV detection at 260 nm (69). Cellular ATP content was measured using an ATPlite kit (PerkinElmer, Milan). Briefly, cells grown in a 48-well plate were killed with 70 μl of luciferase buffer and then 20 μl of D-luciferin was added. The production of light caused by the reaction of ATP with added luciferase and D-luciferin was evaluated within 5 min by means of a luminometer.

### Oxygen consumption analysis

Quantitation of oxygen consumption was conducted by means of the Oxygraph system (Hansatech Instruments) as reported (70). Cells (250,000), exposed or not to NAD+ 1 mM or 100 μM, at different times, were gently detached with trypsin and loaded in the chamber containing 300 μl of DMEM. Oxygen consumption was monitored for 5 min at 37 °C.

### NAD+ and AMP uptake measurement

Cells plated in 12 wells plates were grown to confluence. When cells were preincubated with 1 mM unlabeled NAD+ for 24 h, the wells were washed three times with 1 ml of PBS and then incubated in a buffer solution (140 mM NaCl, 10 mM Hepes/Na, 2.5 mM MgSO_4_, 2 mM CaCl_2_, and 5 mM KCl, 5 mM glucose, pH 7.4) containing 500 pM [^14^C]NAD+ (54 mCi/mmol), [^3^H]NAD+ (28.6 Ci/mmol), or [^14^C]AMP (60 mCi/mmol) (PerkinElmer). Competing compounds and drugs were added to the wells 15 min before labeled molecules unless stated in the text. After 10 min, cells were washed three times with 1 ml of PBS and lysed with 0.5 M NaOH. Incorporated radioactivity was assayed by liquid scintillation counting.

### RNA isolation and qPCR

Total RNA was isolated using TRIzol Reagent (Life Technologies). One microgram of RNA was retrotranscribed using iScript (Bio-Rad). Real-time PCR was performed using Rotor-Gene 3000 (Qiagen) as reported (71). The following primers were used: *NAMPT* forward 5′-AACAATATCCACCCAACACAA-3′, reverse 5′-TAGACATCTTTGGCTTCCTGG-3′; *NMNAT1* forward 5′-TCCCATCACCAACATGCACC-3′, reverse 5′-TGATGACCCGGTGATAGGCAG-3′; *NMNAT2* forward 5′-GATTGGATCAGGGTGGACC-3′, reverse 5′-TCCGATCACAGGTGTCATGG-3′; *NMNAT3* forward 5′-ATGGGAAGAAAGACCTCGCAG-3′, reverse 5′-AGTTTGCTGTGATGATGCCTC-3′; *CD38* forward 5′-CCCGCAGGTTTGCAGAAGCTGCC-3′, reverse 5′-CGATTCCAGCTCTTTTATGGTGGGATC-3′; *CD73* forward 5′-GATATGAGAACTTCTGCTGGAAAGTG-3′, reverse 5′-CAAAACCTCTAGCTGCCATTTGCACAC-3′; *CD157* forward 5′-GTTGCAGATTTCTTGAGCTGGTGTCG-3′, reverse 5′-CCTTTGATGGGATAGGCTCCTGTTGG-3′; *CD203a* forward 5′-CAGATCATGGCATGGAACAAGGCAG-3′, reverse 5′-TGGTTCCCGGCAAGAAAGATTTCGG-3′; *SARM1 forward 5*′*-GCAGTAGCGGTGTTGGCGACTAAC*-*3*′, *reverse 5*′*-TTAGAGTCGAGCAACGGCACGAGG*-*3*′; *NRK1* forward 5′-CAGAAACACCTCCCAAATTGCAGTGTC-3′, reverse 5′-GGGAATTTCCTCAGCACTTTCCTGGT-3′; *Slc12a8* forward 5′-CCATGTATATCACCGGCTTTGCTGAATC-3′, reverse 5′-GAAAGAACCCACCACAAAGTCCAGTG-3′; *SIRT1* forward 5′-CCCAGATCTTCCAGATCCTCAAGC-3′, reverse 5′-GCAACCTGTTCCAGCGTGTCTATG-3′; *SLC29A1* (*ENT1*) forward 5′-TTGCCTGAGCGGAACTCTCTC-3′, reverse 5′-GCATCCAGCTGCACCTTCAC-3′; *SLC29A2* (*ENT2*) forward 5′-TCCTGCAGTCTGATGAGAACG-3′, reverse 5′-GACGGACAGGGTGACTGTGAA-3′; *18S* forward 5′-CGGCTACCACATCCAAGGAA-3′, reverse 5′-GCTGGAATTACCGCGGCT-3’ (IDT Tema Ricerca).

### SIRT1, NAMPT, NRK1, and Slc12a8 silencing

Nine thousand cells were subcultured in 48-well plates and then incubated with 50 nM SIRT1, NAMPT, NRK1, or Slc12a8 siRNA. After 24 h, cells were exposed or not at 1 mM NAD+, and after 24 h, the NAD+ content was measured or RNA was extracted. The following primers were used: SIRT1 5′-CAAAGGAUAAUUCAGUGUCAUGGTT and 5′-AGGUUUCCUAUUAAGUCACAGUACAA; NRK1 5′-GCAGUCACAAUGAAUAAA and 5′-UUGUUGAUUUAUUCAUUG; Slc12a8 5′-CUUCUCAUCAUGUUUGUG and 5′-ACUGUAUCACAAACAUGA (IDT, Tema Ricerca). siRNA for NAMPT was purchased from Qiagen.

### Bioinformatic analyses

Analysis of TF-binding sites in the promoter region of selected genes was conducted by using CiiiDER software (cit. CiiiDER: a tool for predicting and analyzing TF-binding sites). The analysis was performed using the human motifs from the Jaspar 2020 core redundant vertebrate TF-binding site database (http://jaspar.genereg.net/tools). Subsequently, TFs were selected based on their interaction with SIRT1, identified by means of Biological General Repository for Interaction Datasets database.

### Statistical analysis

Data are presented as the mean values ± SD. All differences among groups were performed using ANOVA followed by Tukey's W-test. Levels of significance were *p* < 0.05 (∗), *p* < 0.01 (∗∗), and *p* < 0.001 (∗∗∗). Statistical analyses were carried out using GraphPad Prism (version 7).

## Data availability

All data described in the article are contained within the article.

## Supporting information

This article contains supporting information.

## Conflict of interest

The authors declare that they have no conflicts of interest with the contents of this article.
