# Supplementary figures and images for: SIRT1-dependent restoration of NAD+ homeostasis after increased extracellular NAD+ exposure
Source: J Biol Chem. 2021 Jun 11;297(1):100855. doi: 10.1016/j.jbc.2021.100855 (PMC8233143; doi:10.1016/j.jbc.2021.100855)

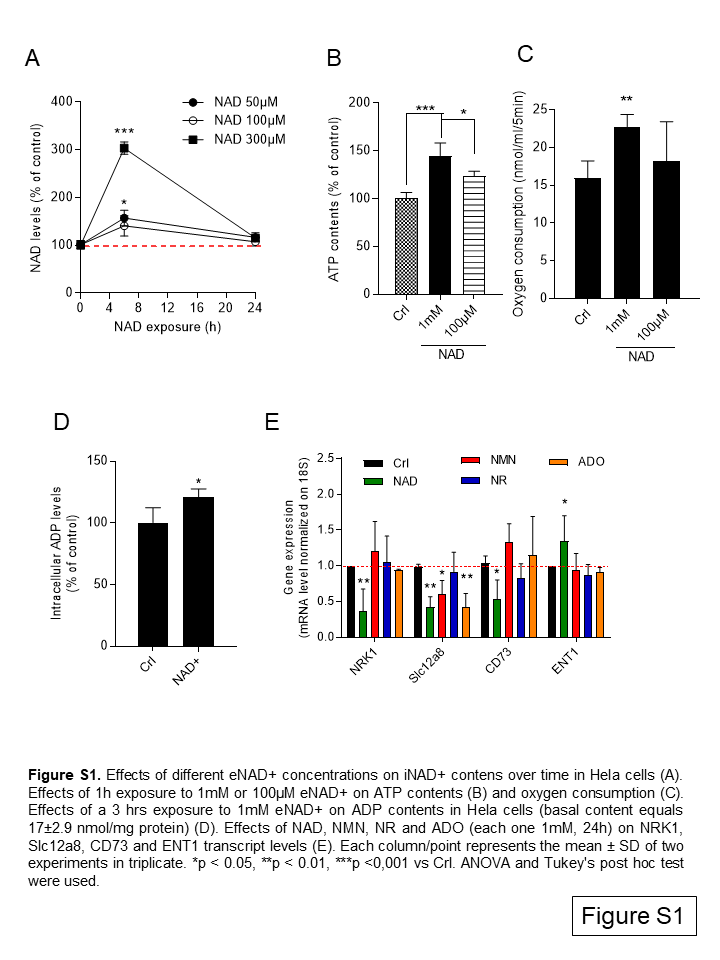


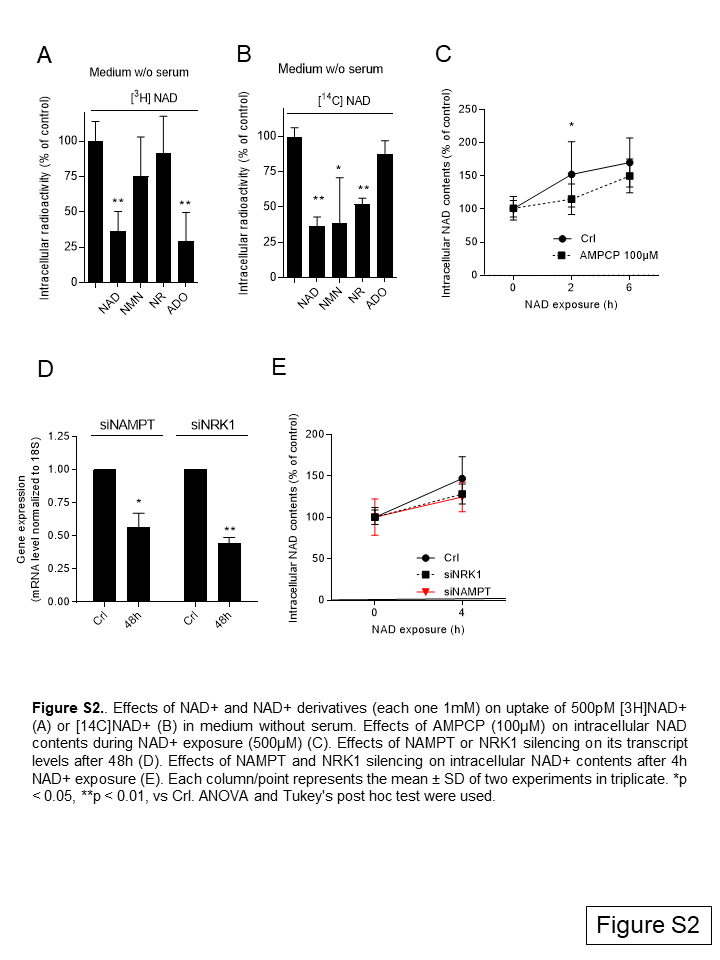

Supplement: Figures S1 and S2 [file mmc1.docx]
